# Description of allergic phenotype in patients with eosinophilic oesophagitis: management protocol proposal

**DOI:** 10.1038/s41598-023-29602-z

**Published:** 2023-02-08

**Authors:** Joan Domenech Witek, Rosario Gonzalez Mendiola, Vicente Jover Cerdá, Javier Pereira González, Clara Carballas Vázquez, Felicitas Villas Martínez, Ramón Rodríguez Pacheco

**Affiliations:** 1 Allergology Unit. H. General Elda, Alicante, Spain; 2 H. Central de La Cruz Roja, Madrid, Spain; 3 H. Quirón Barcelona, Barcelona, Spain; 4 Complejo Hospitalario Universitario A Coruña, A Coruña, Spain; 5 Allergology Unit. Hospital Royo Villanova, Zaragoza, Spain

**Keywords:** Immunological disorders, Gastrointestinal diseases

## Abstract

There is a profile of patient with eosinophilic oesophagitis and atopic background, marked by the existence of IgE-mediated sensitizations. Our aim is to report the observed sensitivities to environmental and food allergens and panallergens in patients with eosinophilic oesophagitis with atopic background as well as characterizing other markers or analytical parameters. We suspect that the prevalence of sensitization to panallergens will be high and this will probably be relevant in terms of the onset and clinical course of the disease. We collated clinical and analytical data from 160 adult patients with a reported diagnosis of eosinophilic oesophagitis. These patients were studied between 1 January 2012 and 31 December 2020. During an initial visit skin tests were performed with full batteries of routine aero-allergens and foodstuffs. Patients were subsequently referred for blood test and determination of specific IgE, blood count and total IgE (in all cases), as well as eosinophilic cation protein and IMMUNOISAC in the centres in which this was available. We were able to detect a broad spectrum of sensitizations to environmental, foodstuffs and panallergens. The most common allergic disease was rhinoconjuntivitis. The sensitizations observed to foodstuffs were atypical for the adult population and were not responsible for manifestations compatible with immediate allergy. An important percentage of patients presented seasonal worsening of choking symptoms. We should be able to identify patients with eosinophilic oesophagitis and atopic background. Identifying this phenomenon would enable giving dietary and environmental recommendations as well as more specific and effective treatments to our patients.

## Introduction

EoE is an inflammatory process involving the oesophageal mucosa and deeper layers. It´s related to food allergy^1,2^. There has been a lot of progress on fundamental aspects such as anatomopathological diagnosis and pharmacological treatments, mainly PPIs (with a response that exceeds 60% of treated patients) and swallowed inhaled corticoids^3^. Another line of treatment in patients with eosinophilic oesophagitis refers to diets, classified into two large groups, empirical (from elementary diets and those to 2, 4 and 6 foodstuffs)^4^ and specific diets, based on performing allergy tests, but with more inconsistent results according to different publications^5^.

Although it has been published that the impact of foods on the clinical course of the disease is not IgE mediated^6^, this has not been proven. The onset and course of EoE probably does not depend on a single mechanism. It is a mixed process where various pathophysiological mechanisms are involved. According to our clinical experience, the existing relationship between development and clinical course of the disease and existence of allergic diseases such as rhinitis, asthma or atopic dermatitis is undeniable. Previous studies have observed the involvement of pollens in these patients^7^. Various publications are in favour or against seasonal worsening of choking symptoms in this patient profile^8^. There are meta-analyses that reveal that the diagnosis of EoE cases is not seasonal and has a normal distribution^9^. Nevertheless different publications talk of an increased existence of eosinophils in the oesophageal mucosa in seasonal periods in patients with allergic rhinitis as well as the impact of perennial allergens in patients responding poorly to diets or cases of development of EoE in regard to administration of sublingual vaccines^10–19^.

However, currently more importance is increasingly given to the impact of panallergens in our daily clinical practice. Among the most prevalent and relevant are LTPs, profilins o TLPs. We know that these panallergens condition significant crossed reactivity phenomena between environmental and food allergies. One characteristic of the allergy to panallergens is that since IgE mediated sensitivities to food is involved, they condition mild symptoms and not necessarily anaphylactic reactions. We must clarify the extent of chronic symptoms and remodelling phenomena of the oesophageal mucosa in EoE patients. They could be related to allergy to panallergens in patients with respiratory allergy to environmental pollens. Previous publications have found statistically significant differences in the prevalence of sensitization to panallergens between patients with eosinophilic oesophagitis and patients with environmental allergy without oesophagitis and healthy controls (*P* < 0.0001)^20^.

In regard to recent bibliography^21^, we believe there is sufficient evidence that justifies in-depth study of EoE patients and possible associated allergic diseases. The problem is the huge variability with which these patients are studied in the different hospitals. Not all are subject to a full allergy study and when this is performed, the tests patients undergo are highly variable. The batteries of allergens used and the determinations of specific IgE are different in each case. The study considered could enable developing a normalized protocol of the study in patients with EoE of atopic profile to detect IgE mediated sensitivities to environmental and food allergens. This protocol would include broad batteries in the case of prick test, determination of specific IgE and if possible, molecular diagnostic techniques and inflammatory mediators.

Since there is a profile of EoE patients with a significant atopic background, associated with allergic diseases, our hypothesis is that if we manage to systematize the allergology study in these patients we will detect a significant number of sensitivities that could be relevant. We also believe there are analytical parameters and biomarkers that may characterize these patients. Finally, we suspect that the prevalence of sensitivity to panallergens will be high and that this is probably relevant in regard to the onset and clinical course of EoE.

Our primary aim was to ascertain the observed sensitivities to environmental and food allergens as well as panallergens in EoE patients with atopic background and characterize other markers or analytical parameters in these patients. We also wanted to ascertain associated clinical and/or sociodemographic factors and observe how many of them with respiratory-like allergy present seasonal worsening of reflux symptoms or food impaction.

## Methods

### Study design

Multicentre, retrospective, descriptive and transversal.

### Ethics aspects

The study did not collate any personal data that can identify the patient and since it was a study that recovered data from the clinical history, no informed consent was necessary (the non-need for informed consent was approved after evaluation by the members of the Ethics Committee of the General Hospital of Elda, with Dr. Julián Izquierdo Luzón as secretary). The study having successfully completed all the requirements imposed by the authorities of this country (including those of the Declaration of Helsinky (WMA, 2008) and Norms of Good Clinical Practice of the European Union) was approved by IRB (Institutional Review Board of Elda General University Hospital). Supporting document is attached.

### Sample selection

To proceed with the study we recovered the clinical and analytical data (clinical history) of adult patients (aged 18 to 80) with reported diagnosis of EoE, in accordance with the currently accepted consensus (15 or more eosinophils by high power field on oesophageal mucosa biopsy). These patients were studied from 1 January 2012 to 31 December 2020 in the five health centres taking part in the study: Hospital General de Elda, Hospital Central de la Cruz Roja de Madrid, Hospital Quirón Barcelona, Complejo Hospitalario Universitario A Coruña and Hospital Royo Villanova, Zaragoza Allergology Unit. During an initial visit and after relevant clinical history skin tests were performed with full batteries of routine aero-allergens and foodstuffs (prick test performed with standardized allergenic extracts from Laboratorios Bial Aristegui/Roxall ®, ALK ® and Leti ®) (Online appendix 1). Patients were subsequently referred for analytical extraction and determination of specific IgE extended to aero-allergens and foodstuffs (Online appendix 2), blood count and total IgE (in all cases), in addition to eosinophilic cationic protein (ECP) and IMMUNOCAP ISAC™ for the sites in which this was available. Over the course of taking the clinical history, patients were asked whether or not they presented seasonal symptoms in addition to whether they had improved with administration of specific immunotherapy. The tests performed correspond to routine clinical practice but only including in the study patients in whom a minimum number of tests had been performed. We considered as positive prick test with diameters equal or greater than histamine and the values of specific IgE greater than 0.35 kUA/L.

### Sample size

Assuming the most unfavourable proportion or *P* = 0.5 (pxq = 0.25) with a 95% confidence interval, n = 160 this corresponds to an approximation of 8%.

### Selection criteria

#### Inclusion criteria

1. Patients with reported diagnosis of EoE referred for study in the allergology departments, sections and units of sites taking part.
2. Patients should have a history of at least one of the following diseases: allergic rhinitis, allergic asthma, food allergy and/or atopic dermatitis.

#### Exclusion criteria

The following cases will be excluded:

1. Patients without a reported diagnosis of EoE.
2. Patients with hyper eosinophilic syndrome.
3. Patients with underlying haematological disorders or immunodeficiencies.
4. Patients in whom it was not possible to perform the full study in accordance with the protocol.

### Statistical study

Descriptive analysis according to type of variable. Proportion for the qualitative variable and central tendency measures (mean, mode and median) and dispersion (typical deviation, standard deviation and variance) for the quantitative variable. Of the most relevant variables 95% confidence intervals will be calculated.

Bivariate analysis, if the variable follows a normal distribution parametric, chi squared tests are used to compare proportions and student *t* tests are used to compare means. If the variable does not follow a normal distribution no parametric tests will be used according to type of variable. Statistical significance is set as *P* < 0.05.

Multivariate analysis, to minimize confounding bias and according to the dependent variable multivariate analysis by binary logistic regression and by steps will be used. Odds ratio with its 95% confidence interval will be calculated as association measure. To evaluate the multivariate model’s discriminative capacity, ROC curves with the areas under the curve and 95% confidence intervals are calculated.

## Results

### Epidemiological data

A total of 160 patients diagnosed with EoE (121 men and 39 women) between January 2012 and December 2020 and aged between 18 and 80 years (37 ± 12.8 years) were included consecutively. The epidemiological variables are summarized in Table 1.

**Table 1:** Clinical and sociodemographic characteristics of the included patients.

Number of patients	n	160
Men/Women	121/39	(76%/24%)
Age	37,4 ± 12,8	
Asthma	44	(27,5%)
Rhinoconjunctivitis	134	(83,75%)
Atopic dermatitis	10	(6,2%)
Aeroallergens	141	(88,12%)
Food allergies	142	(88,75%)
Panallergens sensitization	75	(47%)
Seasonal worsening (n-123 )	22	(17,8%)
Specific diet	98	(89,90%)
Empiric diet	11	(10,10%)
Endoscopic findings		
Stenosing phenotipe	63	(39,37%)
Inflammatory phenotype	97	(60,63%)
Improvement after diet (n-33 )	26	(81,25%)
Analytical values		
Total IgE	383 ± 468,58	
Basal ECP (μg/mL) (n-45 )	39,44 ± 29,5	
Eosinophils Pre diet (periph. blood)	363 ± 237,34	
Patients with Immunoisac	27	(16,8%)
Panallergens sensitization objectified with Isac	19	(70%)

### Clinical data

Most of our patients had food sensitization. We observed an atypical profile of food sensitization in adults, such as allergies to gluten, rice, soy, egg and milk (food sensitizations are summarized in Table 2). The most common atopic feature was rhino-conjunctivitis (83.75%) and there were also cases of asthma (27.5%) and atopic dermatitis (6.2%). We also encountered a number of patients who said their digestive symptoms were worse during respiratory symptom aggravation periods due to pollen exposure (17.8%). Of the total patients, 39.3% presented a stenosing phenotype and 60.63% an inflammatory phenotype in the oesophageal mucosa. A total of 109 patients were offered the possibility of undergoing treatment with diets. Of these, 98 (68.75%) maintained specific diets based on allergy tests and 11 (27.5%) empirical diets of six foodstuffs. Endoscopy monitoring could be performed after the procedure in 48 of these cases. Of these, 32 controls corresponded to patients treated with specific diets, observing anatomopathological improvement in 26 (81,25%) cases and no significant improvement in 6 (18,75%).

**Table 2:** Objectified food sensitizations.

Food	Number of patients sensitizied
Nuts	80 (50%)
Fruits	72 (45%)
Gluten	65 (41%)
Milk	49 (31%)
Soy/Legumes	47 (29%)
Egg	35 (22%)
Fish	29 (18%)
Seafood	22 (14%)
Solanaceae	22 (14%)
Corn	18 (11%)
Meat	12 (7,5%)
Rice	11 (7%)
Anisakis	4 (2,5%)
Cocoa	2 (1,2%)

### Analytical values

At baseline, all our patients presented with high ECP levels compared with the normal accepted standard^22^, with average levels of 39.44 μg/mL ± 29.5 (n 45). We measured a basal peripheral blood eosinophil count of 363 ± 237.34. IgE measured was 383 mg/dL ± 468.58.

The food allergens most commonly observed were nuts (50%), fruits (45%), gluten (41%) and milk (31%). The full list of sensitizations observed can be seen in Table 2. The aero-allergens observed with most prevalence were grass (57% of cases), dust mites (44%), epithelia (42.5%) and olive (40%). The remaining sensitizations observed can be consulted in Table 3.

**Table 3:** Objectified sensitizations to aeroallergens.

Aeroallergen	Number of patients sensitizied
Poaceae	91 (57%)
Dust mite	70 (44%)
Epitelium	68 (42,5%)
Olea	64 (40%)
Amarantaceae	39 (24%)
Platanus	28 (17,5%)
Alternaria	24 (15%)
Plantago	23 (14%)
Cypress	23 (14%)
Artemisia	21 (13%)
Parietaria	18 (11%)

Detailing the results according to age groups, we observe that the most prevalent foods in the patient group aged under 20 were nuts, fruits, gluten and soya/legumes. In the group aged 20 to 40 and 40 to 60 the most prevalent foods were fruits, gluten, milk or soya/legumes. In the group aged over 60, the most prevalent were gluten and milk. No significant differences were observed in regard to sensitization to environmental aero-allergens with pollen from grass, epithelia, mites and olive pollen the most prevalent. If we differentiate by sexes, sensitization was similar in men and women although we observe differences in regard to those most prevalent in each case. For men, the most common were nuts, gluten, fruits and soya and in women, by order of prevalence they were fruits, gluten, nuts and milk.

The high prevalence of sensitization to panallergens observed in 47% of cases is highly notable. In detail, we observed sensitization to LTP, profilin and TLPs in 27.5%, 25% and 2.5% of patients, respectively. Polysensitization to panallergens was observed in 6.25% of cases. We highlight that using specific molecular diagnostic techniques (IMMUNOISAC) the prevalence of sensitization to panallergens attained 70% (19 of 27 performed).

Finally, we observed that sensitization to panallergens in cases of allergy observed to vegetable origin food allergens was very high. A total of 100% of patients with allergy to solanaceae were sensitized to panallergens. This prevalence of sensitization was observed in 94% of patients with fruit allergy and 75% to 89% of patients with allergy to gluten, soya, rice and dry fruits.

## Discussion

We present one of the longest databases focused on the study of patients with allergic phenotype EoE. We reveal that by standardizing the tests performed both in vivo and in vitro and working with a broad spectrum of allergen detection, we can observe numerous sensitivities both to food and environmental allergens and panallergens. Our protocol is based on use of very broad batteries in all patients with no limit on tests performed based on the triggering factors reported in the clinical history.

As in previous publications we believe that there are multiple phenotypes of eosinophilic oesophagitis and that essentially this is a multifactorial condition. Factors have been published that confirm there is an allergic phenotype in these patients. For example an increased seasonal population of eosinophils in the oesophageal mucosa has been observed in patients with allergic rhinitis. Proteases from dust ticks can affect the correct function of the oesophageal mucosa^23^. We believe that in the same way as treatments with sublingual immunotherapy were able to lead to EoE, exposure to environmental aero-allergens must be involved in the onset and development of this disease. In our study the prevalence of seasonality is lower than expected (17.8%). This may be due to an underestimate because of the difficulties questioning patients in some groups in person because of the current pandemic. For groups in which this has been done in detail the prevalence of seasonality attains 36%.

One of the most notable findings in our study is the high prevalence observed for sensitization to panallergens. We should consider that not all investigators taking part have been able to use specific diagnostic techniques and that probably the prevalence of sensitization is higher than that discussed in Table 1. In fact, the percentage sensitization observed if we use molecular diagnostic techniques is high, attaining up to 70% of the total of those performed. We are increasingly aware of the importance of molecular diagnostic techniques for sensitization to panallergens in regard to pollen-foodstuff crossed reactivity syndromes. It has been reported that these syndromes may be related to symptoms that range from oral food allergy syndrome to anaphylaxis^8^.

In conclusion, allergic phenotype eosinophilic oesophagitis is a condition that mainly affects males average aged 37 years with a history of rhinoconjuntivitis and/or asthma. Analytically they present high levels of ECP (39 mcg/mL), eosinophilia higher than 300 absolute eosinophils and total IgE higher than 300 mg/dL. More prevalent food allergies were nuts, fruit, gluten, milk and environmental allergens to pollens (grass and olive) and animal epithelia. The prevalence of sensitization to panallergens (LTP and profilin) is notable. Nonetheless we must consider that sensitizations may depend in large part on good habits and the flora of the patient’s surrounding. It would be interesting to ascertain the prevalence of sensitization to panallergens in other allergic pathologies. Similar results have been observed in previous publications with a high prevalence of sensitization to pollens, foods (nuts) and panallergens. In these patients, clinical improvement was observed in 78.3% of the cases with anatomopathological resolution in 75% of the cases, after the indication of specific diets and immunotherapy with allergens^24^.

We propose the development and implementation of a standardized study protocol (Online appendix 3) for patients with atopic EoE. This protocol would be based on systematically performing very broad batteries of aero-allergens and foodstuffs and for the use of molecular diagnostic techniques (IMMUNOCAP ISAC™) to reveal sensitization to panallergens and identify pollens-foodstuffs crossed reactivity syndromes. This would enable establishing specific diets based on the results of the proposed study. In our next publication we will seek to validate the effectiveness of these diets in an atopic profile of patients with EoE, by performing endoscopic and anatomopatholygic controls after compliance with them.

## Limitations of the study

A large part of the project was performed in the context of the global COVID-19 pandemic. This has meant a limitation when citing patients in person to repeat or extend certain tests, whereby not all data are available for all patients. Nonetheless, the criteria deemed basic were compliant in all cases. When data were not obtained for all patients (ECP or INMUNOISAC, for example), this has been clearly reflected in the “n” results section for each case.

## Supplementary Information

Supplementary Information 1.

Supplementary Information 2.

Supplementary Information 3.

## Data Availability

All the clinical and laboratory data are available, on request, at joanwitek@yahoo.es in the respect of patients’ anonimity.

## Abbreviations

EoE: Eosinophlic oesophagits
ECP: Eosinophilic cationic protein
LTP: Lipid transporter protein
PPI: Proton pump inhibitor
TLP: Thaumatin-like protein
