# Supplementary material for: Description of allergic phenotype in patients with eosinophilic oesophagitis: management protocol proposal
Source: Sci Rep. 2023 Feb 8;13:2226. doi: 10.1038/s41598-023-29602-z (PMC9906574; doi:10.1038/s41598-023-29602-z)
Supplement: Supplementary file 1 — Supplementary Information 1. [file 41598_2023_29602_MOESM1_ESM.pdf]

APPENDIX 1: PRICK TEST PERFORMED

AEROALLERGENS:

D. pteronyssinus
D. farinae
Cynodon dactilon
Poaceae
Dactylis
Artemisia vulgaris
Chenopodium album
Parietaria judaica
Plantago ovata
Salsola kali
Cupressus arizonica
Olea europaea
Pinus pinea
Platanus acerifolia
Profilin
Alternaria alternata
Aspergillus fumigatus
Cladosporium herbarum
Botrytis cinerea
Penicillium chrysogenum
Blatella germanica
Cat epithelium
Dog epithelium

FOOD STUFF:

Anisakis simplex
Latex
Strawberry
Banana
Avocado
Melon
Apple
Plum
Peach
Kiwi
Grape
Almond
Hazelnut
Chesnut
Pistachio
Sunflower seeds
Nut
Peanut
Rice
Gluten
Wheat
Soy
Gliadin
Peppers spice
Sesame
Mustard
Egg/Related proteins.
Milk/Related proteins
Whitw/blue fish
Tuna
Seafood
Clam
Squid
Garlic
Pepper
Tomato
