# Supplementary material for: Description of allergic phenotype in patients with eosinophilic oesophagitis: management protocol proposal
Source: Sci Rep. 2023 Feb 8;13:2226. doi: 10.1038/s41598-023-29602-z (PMC9906574; doi:10.1038/s41598-023-29602-z)
Supplement: Supplementary file 2 — Supplementary Information 2. [file 41598_2023_29602_MOESM2_ESM.pdf]

APPENDIX 2

MINIMUM SPECIFIC IgE PERFORMED

<u>AEROALLERGENS:</u>

D. pteronyssinus
D. farinae
Dog epithelium
Cat epithelium
Cynodon dactilon
Dactylis glomerata
Salsola kali
Chenopodium album
Olea euruopaea
Alternaria alternata
Cupressus arizonica
Platanus acerifolia

<u>FOOD STUFF:</u>

Peach
Melon
Kiwi
Banana
Peanut
Nut
Almond
Rice
Soy
Blue/white fish
Tuna
Seafood
Clam
Squid
Anisakis simplex
Milk
Alfa-lactoalbumin
Beta-lactoglobulin
Casein
Egg/proteins.
Tomato
Sesame
Mustard
Anisakis simpleax
Gluten/wheat
Latex
