# Supplementary material for: Description of allergic phenotype in patients with eosinophilic oesophagitis: management protocol proposal
Source: Sci Rep. 2023 Feb 8;13:2226. doi: 10.1038/s41598-023-29602-z (PMC9906574; doi:10.1038/s41598-023-29602-z)
Supplement: Supplementary file 3 — Supplementary Information 3. [file 41598_2023_29602_MOESM3_ESM.pdf]

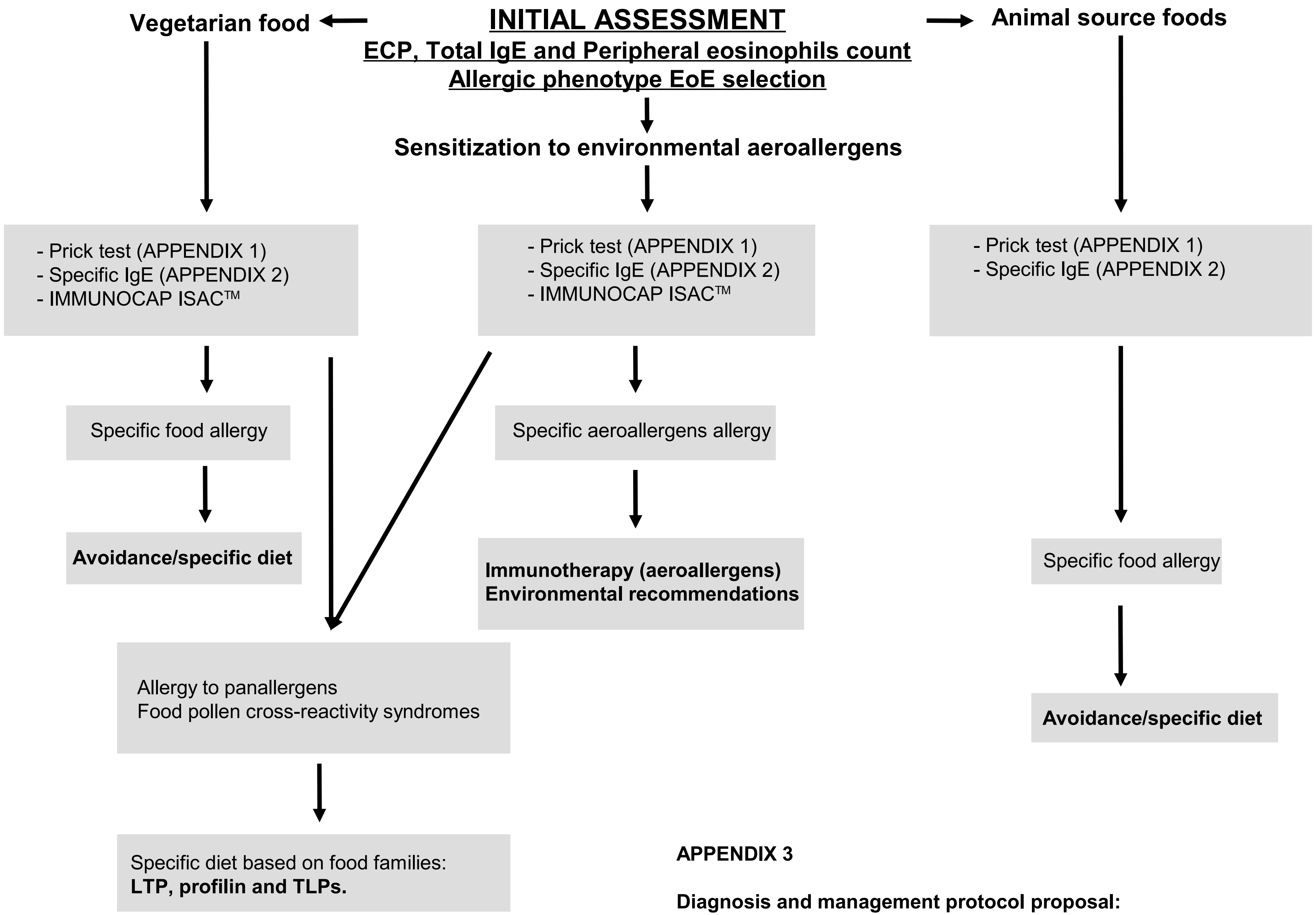

APPENDIX 3

Diagnosis and management protocol proposal:
Performing endoscopic controls after diets and
evaluation with quality of life questionnaires would be imperative
